# Temperature-dependent changes in neuronal dynamics in a patient with an *SCN1A* mutation and hyperthermia induced seizures

**DOI:** 10.1038/srep31879

**Published:** 2016-09-01

**Authors:** C. Peters, R. E. Rosch, E. Hughes, P. C. Ruben

**Affiliations:** 1 Department of Biomedical Physiology and Kinesiology, Simon Fraser University, Burnaby, BC, Canada; 2 Wellcome Trust Centre for Neuroimaging, Institute of Neurology, University College London, UK; 3 Centre for Developmental Cognitive Neuroscience, Institute of Child Health, University College, London, UK; 4 Department of Paediatric Neurology, Evelina London Children’s Hospital, Guy’s and St Thomas’ NHS Foundation Trust, London, UK

## Abstract

Dravet syndrome is the prototype of *SCN1A*-mutation associated epilepsies. It is characterised by prolonged seizures, typically provoked by fever. We describe the evaluation of an *SCN1A* mutation in a child with early-onset temperature-sensitive seizures. The patient carries a heterozygous missense variant (c3818C > T; pAla1273Val) in the Na_V_1.1 brain sodium channel. We compared the functional effects of the variant *vs*. wild type Na_V_1.1 using patch clamp recordings from channels expressed in Chinese Hamster Ovary Cells at different temperatures (32, 37, and 40 °C). The variant channels produced a temperature-dependent destabilization of activation and fast inactivation. Implementing these empirical abnormalities in a computational model predicts a higher threshold for depolarization block in the variant, particularly at 40 °C, suggesting a failure to autoregulate at high-input states. These results reveal direct effects of abnormalities in Na_V_1.1 biophysical properties on neuronal dynamics. They illustrate the value of combining cellular measurements with computational models to integrate different observational scales (gene/channel to patient).

Epilepsy is a common neurological condition, with particularly high incidence in early childhood^1^. The diagnosis is made when patients show continued susceptibility for recurrent epileptic seizures. Although in some patients this susceptibility is due to structural brain abnormalities, including tumours, previous strokes, or congenital malformations, for a majority the suspected cause is genetic^2^.

Specific mutations in a wide variety of ion channel and synaptic genes have been found to cause epilepsy in individual patients and families^3^. Somewhat surprisingly, many of these mutations were identified not from common, familial epilepsies (recently renamed *genetic generalised epilepsies*)^4^, but from epileptic encephalopathies^5^, sporadic severe epilepsies occurring mainly in childhood.

One of the first ion channel “epilepsy” genes identified was *SCN1A*, which encodes the neuronal voltage-gated sodium channel Na_V_1.1. Initially described in patients with Generalised Epilepsy with Febrile Seizures plus Type 2^6^, it is also the dominant genetic cause of Dravet syndrome, previously termed severe myoclonic epilepsy of infancy^7^, an early onset epileptic encephalopathy. The link between the two conditions was made because of the shared susceptibility to recurrent, prolonged febrile seizures; patients living with Dravet syndrome in particular experience seizures in response to increases in environmental temperatures, such as hot baths^8^.

Mammalian voltage-gated sodium channels are comprised of 4 domains (DI–DIV) encoded by a single transcript. Each domain contains 6 transmembrane segments (S1–S6)^9^; S1–S4 form the voltage sensing region and S5 and S6 form the pore forming region. (Fig. 1). The movement of the positively charged S4s to the extracellular side of the membrane leads to opening of the channel pore and an influx of sodium^10^,^11^. Following channel activation, the current ceases due to a process termed fast inactivation in which the linking segment between DIII and DIV moves to occlude the channel pore (illustrated as *h* in Fig. 1)^12^. Channel missense mutations which lead to channels with altered rates and voltage dependence of gating can impair electrical signalling and, in the case of neuronal channels, lead to different epilepsy syndromes.

*SCN1A* mutations related to Dravet syndrome include severe disruptions of channel integrity (e.g. frameshift mutations, deletions), and, less commonly, missense mutations leading to either channel impairment or gain of function. The reported prevalence of loss-of-function mutations in clinical cohorts^13^ may be counterintuitive, since they would render neurons less excitable and thus less seizure-prone. This paradox may be explained by studies suggesting Na_V_1.1 channels are predominantly found in GABAergic interneurons, where loss-of-function may cause overall cortical disinhibition, permissive of epileptic activity^14^,^15^. This also explains the paradoxical exacerbation of seizures in response to sodium channel blocking antiepileptic drugs observed in many Dravet patients^16^.

This Na_V_1.1 haploinsufficiency account of epileptogenesis in Dravet and associated epilepsies does not fully explain all clinical observations. A significant number of patients with gain-of-function mutations show severe epilepsy phenotypes considered more typical of deletion or frameshift mutations^13^,^17^,^18^. Mapping possible direct mechanistic links between sodium channel mutations and increased seizure susceptibility may help improve our understanding of genotype-phenotype correlations. By integrating experimental measurements into a computational model of neuronal function, we can predict the effects of mutations on neurons *in silico*. This may identify otherwise unpredictable functional effects of genetic mutations and group abnormalities in the system into functionally relevant categories^19^,^20^.

We present a patient with an early-onset, temperature-sensitive epilepsy phenotype and a heretofore uncharacterised *de novo* heterozygous *SCN1A* mutation (c.3818C > T, ClinVar Accession: RCV000180969.1) coding for a mutant in DIIIS2 of the Na_V_1.1 channel (p.Ala1273Val). Using patch-clamp characterisation of channel properties, we identify dynamic, temperature-dependent differences from wild type (WT). Integrating these empirical results in computational models of action potential dynamics at the membrane of a cortical neuron, we specify the functional effects of the mutation and describe a mechanism that leads to temperature-sensitive epilepsy.

## Clinical Case Report

This child was first admitted at the age of 6 months with a brief, self-terminating febrile seizure with a right-sided predominance of his twitching movements. He subsequently presented with prolonged recurrent seizures, both with and without fever, some lasting 30 minutes or more. These seizures required emergency treatment with benzodiazepines, and one intensive care unit admission related to respiratory depression following treatment, as a consequence of which treatment with phenytoin was commenced, which reduced the duration of his seizures to less than 5 minutes. Interestingly, a proportion of these seizures were apparently provoked by a hot bath, or whilst playing in a very warm environment.

There was no evidence of focal neurological impairment after recovering from seizures during any of his hospital admissions. He was born at term and had an uncomplicated perinatal course. There was no family history of epilepsy, neurodevelopmental or psychiatric conditions. No abnormalities were found on systemic examination and extensive cardiology review; his echocardiogram and electrocardiogram were unremarkable.

Because of the clinical phenotype he underwent genetic sequencing of the *SCN1A* gene at 12 months of age. This showed a *de novo* heterozygous missense mutation (c.3818C > T) causing changes in a functionally significant and highly conserved region of the *SCN1A* protein (p.Ala1273Val). This genetic mutation, together with the clinical context suggests a diagnosis of a seizure disorder within the wider Dravet Syndrome spectrum.

Following his genetic diagnosis, his treatment was changed to sodium valproate, which he tolerates well and which has markedly reduced the number and duration of seizures. At the current time he continues to make age appropriate developmental progress.

## Results

Data from experiments performed at 32 °C can be found in the supplementary material (Supplementary Table S1) Source data for all figures including means, standard errors, and number of individual experiments (N) can be found in the supplementary material (Supplementary Tables S2–S5).

### Na_V_1.1 Activation

**Figure 2:**
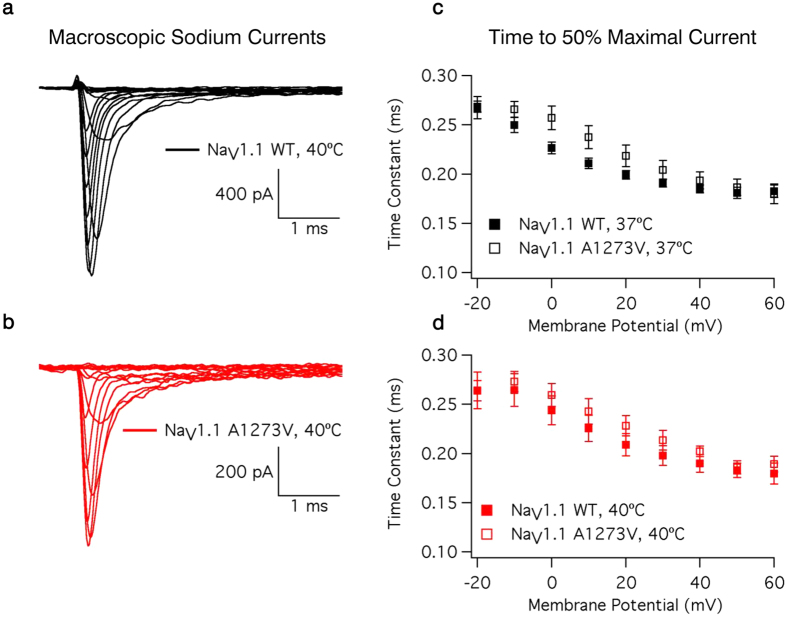
Macroscopic Na_V_1.1 Currents. Sample WT (**a**) and A1273V (**b**) currents elicited at potentials between −100 mV and +60 mV at 40 °C. Time to 50% maximal current is plotted versus voltage for WT and A1273V Na_V_1.1 channels at 37 °C (**c**) and 40 °C (**d**). Source data for time to 50% maximal current can be found in Supplementary Table S2.

**Figure 3:**
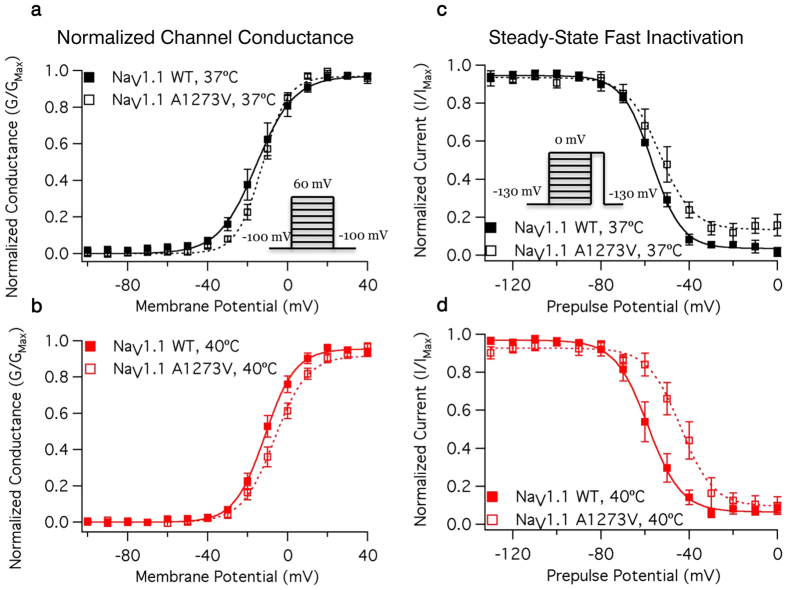
Voltage Dependence of NaV1.1 Conductance and Fast Inactivation. Normalized Conductance curves for WT and A1273V Na_V_1.1 channels at 37 °C (**a**) and 40 °C (**b**). Conductance was determined from macroscopic current recordings using Ohm’s law corrected for the experimentally observed equilibrium potential. Normalized current during a test pulse following a 200 ms pre-pulse is plotted versus pre-pulse potential for WT and A1273V Na_V_1.1 channels at 37 °C (**c**) and 40 °C (**d**). Pulse protocols used to elicit macroscopic current and to determine the voltage-dependence of fast inactivation are shown in the insets of **a** and **c**, respectively. Source data for the voltage dependence of activation and fast inactivation can be found in Supplementary Table S3.

Sample macroscopic sodium currents from WT and A1273V channels are shown in Fig. 2a,b, respectively. There is no significant difference in the time to 50% maximal current between WT and A1273V channels (from −20 mV to +60 mV in 10 mV intervals: P = 0.7681, 0.1564, 0.0803, 0.0896, 0.0749, 0.1743, 0.4456, 0.2645, 0.5020) nor is there a difference in temperature sensitivity (From −20 mV to +60 mV in 10 mV intervals: P = 0.8318, 0.8101, 0.3128, 0.3882, 0.3936, 0.4212, 0.3160, 0.5845, 0.2824) (Fig. 2c,d, Supplementary Table S2). Increasing temperature significantly accelerates the time to 50% maximal current at potentials between −20 mV and +50 mV (from −20 mV to +60 mV in 10 mV intervals: P = 0.0006, 0.0046, 0.0047, 0.0061, 0.0077, 0.0127, 0.0149, 0.0215, 0.1239) in both WT and A1273V. There is a significant difference in temperature sensitivity of the conductance-voltage relationship between WT and A1273V (P = 0.0235) (Fig. 3a,b, Supplementary Table S3). At 37 °C (Fig. 3a) the A1273V curve is depolarized by only 2.1 mV compared to WT while at 40 °C (Fig. 3b) this difference is increased to 5.8 mV.

### Na_V_1.1 Fast Inactivation

There is a significant difference in the temperature sensitivity of the midpoint of steady-state fast inactivation between WT and A1273V (P < 0.0001) (Fig. 3c,d, Supplementary Table S3). At 37 °C the A1273V steady-state fast inactivation midpoint is depolarized by 1 mV compared to WT (Fig. 3c); the difference increases to 14 mV at 40 °C (Fig. 3d). The fast time constant of recovery is significantly accelerated by increases in temperature (P = 0.0028); the slow time constant of recovery is not (P = 0.8631). Neither the fast nor slow time constants of recovery are altered by the mutation (P = 0.8229 and 0.0828, respectively).

**Figure 4:**
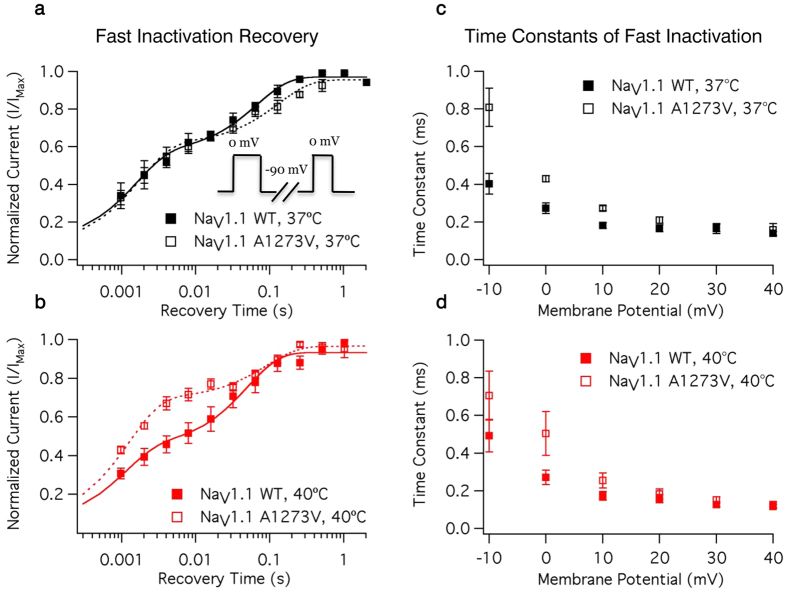
Time Course of Fast Inactivation. Open-state fast inactivation time constants are plotted versus voltage for WT and A1273V Na_V_1.1 at 37 °C (**a**) and 40 °C (**b**). The time course of fast inactivation recovery versus recovery is plotted for WT and A1273V Na_V_1.1 at 37 °C (**c**) and 40 °C (**d**). The double-pulse protocol used to measure fast inactivation recovery is shown in the inset of **a**. Source data for fast inactivation time constants can be found in Supplementary Tables S4 and S5.

There is a significant difference in the temperature sensitivity of the recovery component amplitudes in the A1273V mutant compared to WT (P = 0.0021). Increasing temperature decreases the fast component amplitude and increases the slow component amplitude in WT channels; the opposite occurs in A1273V. The overall results are an increased recovery in A1273V (Fig. 4b) channels at 40 °C compared to WT (Fig. 4a) channels (Supplementary Table S4). Open state fast inactivation time constants are shown for WT and A1273V channels in Fig. 4c,d, respectively. A mutant effect on fast inactivation onset occurs only at 0 mV (from −10 mV to +40 mV in 10 mV intervals: P = 0.1066, 0.0366, 0.0734, 0.6507, 0.4492, 0.5990) (Supplementary Table S5). As we tested for effects at 6 voltages and only found evidence of a difference at 1 voltage, we conclude that A1273V has minimal impacts on fast inactivation onset.

**Figure 5:**
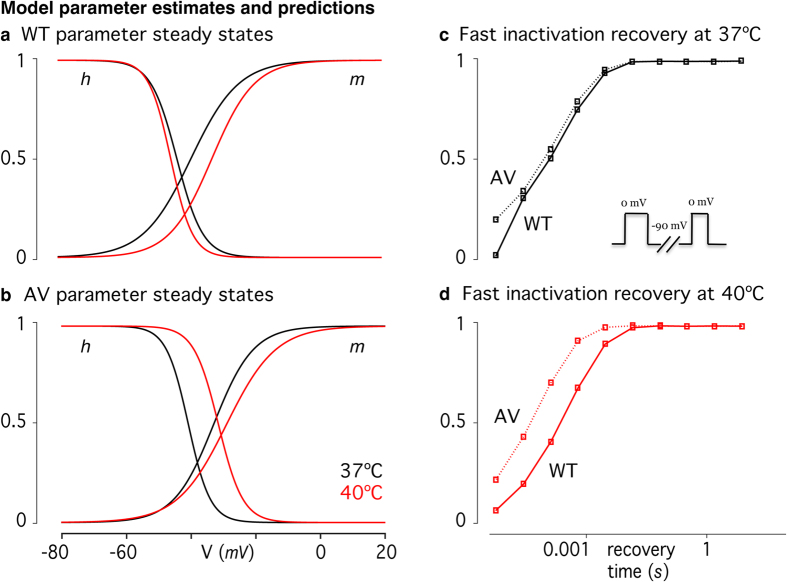
Computational Model Fits to Experimental Data. Steady state values for different voltages were estimated for both the *m* and the *h* parameters based on values from cortical Hodgkin-Huxley-type neuronal simulations. A Boltzmann formulation of the steady state equation was then fitted to the estimates to derive baseline parameter values for the slopes (s_m_ and s_h_) and half-peak voltages (V_2m_, V_2h_). Experimental results from voltage clamp experiments were then translated into changes from these baseline parameters to produce steady state curves for (**a**) the WT, and (**b**) the mutation gating parameters at different temperatures. Forward and reverse rates of the fast inactivation gate (α_h_(V) and β_h_(V)) were shifted along the voltage axis to correspond to the shifts in half-peak voltage of steady-state fast inactivation. The resultant recovery time courses for fast inactivation are shown for WT and the mutant at 37 °C (**c**) and 40 °C (**d**).

**Figure 6:**
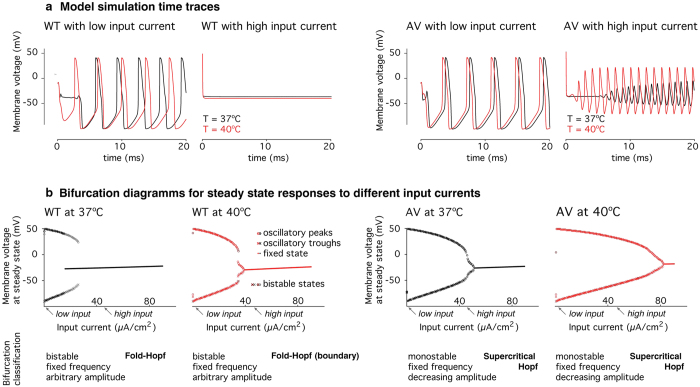
Computational Modelling of Membrane Dynamics. Experimental voltage clamp measurements for four experimental conditions (WT and A1273V at 37 °C and 40 °C) were integrated into a Hodgkin-Huxley model of cortical neurons and normalised to the WT measurements at 37 °C. (**a**) Simulations of the membrane response at different input current levels revealed absent action potential generation in the WT at high currents, even when A1273V neurons continue to fire. (**b**) Bifurcation analysis shows differences in the transition from oscillation to fixed steady states at high input currents (i.e. depolarisation block), both qualitatively in terms of the bifurcation type, and quantitatively in terms of the input currents required to achieve depolarisation block. Of all experimental conditions modelled, A1273V neurons permit the highest input currents to elicit continuous action potentials. Model parameters: *g*_*L*_ = *2.5*  *  *10*^*−5*^ *S*/*cm*^*2*^, *E*_*L*_ = −*70.3*, *g*_*Na*_ = *0.056 S/cm*^*2*^, *E*_*Na*_ = *50*–*mV*, *g*_*K*_ = *0.005 S/cm*^*2*^, *E*_*K*_ = −*90* *mV*, *V*_*t*_ = −*60* *mV*, *C*_*m*_ = *0.01* *μF/mm*^*2*^ 39; high input current *I*_*stim*_ = *45* *μA/mm*^*2*^, low input current *I*_*stim*_ = *0.2* *μA/mm*^*2*^. Remaining parameters were condition specific and defined as described in the Methods section.

We implemented cortical neuron models incorporating WT and A1273V data at 37 and 40 °C, which incorporates the observed shifts in gating parameters *m* and *h* (Fig. 5a,b), as well as the observed mutant effect in fast inactivation dynamics (Fig. 5c,d). Simulations with minimal input (*I*_*stim*_ = *0.2*, Eq. 1.1) reveal little difference in firing frequency or amplitude for either temperature condition (Fig. 6a). As the input stimulus increases, the action potential firing rates of WT models increases faster than those of A1273V models (Supplementary Figure S1). The lowest firing rate at a given stimulus is that of the A1273V model at 40 °C.

### Cortical Neuron Modelling

Simulations with high input currents (*I*_*stim*_ = *45μA/mm*^2^, Eq. 1.1) reveal significant divergence in the dynamic behaviours of different model neurons. Although there is no response from the WT neurons at either temperature (due to depolarisation block), there is continued action potential firing in A1273V neurons at both temperatures, with higher amplitude firing at 40 °C.

Transitions between fixed steady states and oscillations (bifurcations) characterise the dynamic behaviour of neuronal oscillators^21^. Comparing A1273V to WT, there is a shift of the oscillation offset bifurcation towards higher input current values, particularly at 40 °C (Fig. 6b). This means A1273V neurons in the hyperthermic condition continue to produce action potentials at very high input currents. The model also shows differences in firing frequency – with slower firing in the A1237V neurons for any given input current. (Supplementary Figure S1).

## Discussion

This study highlights the value of characterising *SCN1A* variants at elevated temperatures. One previous study identified a temperature-sensitive loss-of-function mutant, R865G, in the Na_V_1.1 channel^22^. R865G leads to primarily gain-of-function properties in channel gating at 37 °C with depolarization of steady-state fast inactivation, hyperpolarization of channel conductance, and increased window current. At 40 °C the mutant also leads to a decrease in channel current during repetitive depolarizations. Most functional studies sodium channel function in Dravet syndrome and related epileptic disorders has focussed on animal models of human *SCN1A* mutations (including fruit flies^23^, zebrafish^24^, and mice^25^). These models are very flexible in that they can be used to investigate a range of genetic changes (including, e.g. heterozygous knockouts), and produce whole-organism phenotypes (including neuronal microcircuitry abnormalities, excitation-inhibition imbalance, and behavioural febrile seizure phenotypes). However, it is often not possible to fully characterise sodium channel gating parameters and their temperature dependence. Furthermore, the isolated contributions of channel-, cell-, and network-level abnormalities as possible routes from genotype to phenotype can not be disentangled with whole-organism models alone.

Recently, a model of patient-derived induced pluripotent stem cells (iPSCs) has also been developed and suggests that patient derived pyramidal cells show evidence of hyperexcitability in isolation (i.e. even in the absence of faulty GABAergic control)^26^. Building on this background, our study further suggests that a single mutation can confer both temperature-sensitive gain-of-function (i.e. action potential generation at higher input currents) and loss-of-function (i.e. lower action potential frequency) in Dravet syndrome.

The A1273V mutant investigated here occurs in the domain III voltage-sensor, specifically near the intracellular side of S2. The movement of the DIII voltage-sensor is part of the activation of the sodium channel^10^. Furthermore, DIII may play a role in channel fast inactivation. Previous work suggests that the primary determinant of channel inactivation is the movement of DIVS4 followed by the binding of the DIII-DIV linker to the intracellular side of the channel^12^,^27^; however, as the movements of DIII are immobilized by channel inactivation, it may also play a role in the binding of the DIII-DIV linker^28^. Thus mutants which impact the movement of DIIIS4 may affect both channel activation and fast inactivation.

In the case of A1273V, we show destabilization of both channel activation and fast inactivation at elevated temperatures. As temperature is elevated to 40 °C the WT channels show relatively little temperature dependence with shifts of +4.1 and −1.9 mV in the conductance-voltage relationship and steady-state fast inactivation, respectively. In contrast, the A1273V mutant is shifted by +8.0 and +9.3 mV in the conductance-voltage relationship and steady-state fast inactivation, respectively. These data suggest that the mutant DIII voltage-sensing domain may be stabilized in the inward conformation. This stabilization is unmasked at elevated temperatures. Crystal structures are not available for the voltage-sensing domains of mammalian sodium channels, limiting our ability to give a molecular mechanism for the impacts of A1273V on Na_V_1.1. However, we hypothesize that the presence of a bulkier valine near the intracellular side of the DIII voltage sensor impedes and slows the outward rate of DIIIS4. This slowing becomes more apparent at elevated temperatures when presumably the rates of the other three voltage-sensors are accelerated. It is also possible that the mutant alters the balance of the inward and outward rates of DIIIS4, which may lead to a decreased probability of activation at elevated temperatures. This may explain why at 40 °C a more depolarized voltage could be required to activate the DIIIS4 which in turn would depolarize the opening of the channel pore.

Our data provide further support that DIII is part of the fast inactivation machinery in the channel. The WT channels undergo a small hyperpolarizing shift in fast inactivation as temperature is increased from 37 °C to 40 °C. A larger hyperpolarization is apparent if the temperature range studied is increased to 32 °C to 40 °C (Supplementary Tables S1 and S3). This would be consistent with DIVS4 being stabilized in the outward conformation as temperature is increased and a decreased availability of channels, which may limit high frequency firing. In A1273V there is a depolarizing shift in the fast inactivation between 32 °C and 40 °C (Supplementary Tables S1 and S3). This suggests that a destabilization of the outward state in DIIIS4 can destabilize fast inactivation possibly through either decreased availability of a DIII binding site for the fast inactivation particle, or inter-domain interactions of the voltage sensors which have been previously shown^29^. In conjunction with the increased rate of channel recovery, the increased availability of sodium channels in the mutant at 40 °C may allow for higher neuronal firing rates, consistent with the decrease in depolarization block in our models. We found evidence of an effect on the rate of open-state inactivation at only one voltage (0 mV). As this was one of the least positive potentials at which we measured open state inactivation, we conclude that at more depolarized voltages the mutant does not exert large effects on the rate of inactivation, but this may not be true at more negative voltages.

Our findings show functional impairments of A1273V are temperature-specific, suggesting a direct link between the disease phenotype and the genetic mutation. Embedding these functional abnormalities at the channel level into a computational neuronal model suggests that, while low input stimulation leads to decreased action potential firing, the maximum stimulus current which results in oscillations is higher in the mutant at 40 °C.

The modelling results are directly correlated to our empirical measurements. Decreased action potential firing is consistent with an increased threshold for action potential firing, predicted by the depolarized conductance-voltage relationship, which is a loss of function in the mutant channel. The lack of depolarization block, in contrast, is consistent with the depolarization in the fast inactivation relationship, which is a gain of function leading to increased channel availability at a given resting membrane potential. Depolarisation block describes the observation that neurons stop responding at high levels of stimulation despite being depolarised beyond the firing threshold. There is evidence that it might play a role in seizure termination^30^,^31^. The difference in depolarisation block thresholds for the different models corresponds to the empirically measured changes in channel gating parameters: Specifically, in AV40 both channel gates are shifted to more depolarized potentials allowing oscillations to occur at more depolarised potentials.

Our model also suggested some more subtle differences. In the WT, there are bistable bifurcations in which neurons that enter depolarisation block are subsequently more likely to remain at a fixed (i.e. blocked) steady state when stimulating currents decrease. In models incorporating the mutant channel dynamics, bifurcations are monostable; neurons which enter depolarisation block return to oscillatory behaviour when stimulation falls even just below the bifurcation threshold. These findings suggest a pathophysiological mechanism in which neurons recover their excitability more quickly after high frequency stimulation.

The change between these different oscillation-offset bifurcation types occurs gradually and the bistability can be seen to collapse into a monostable bifurcation across the different conditions (Fig. 6B). This observation likely relates to interactions between dynamic behaviours of the different voltage gated parameters in the full Hodgkin-Huxley model: through changes in the sodium channel gating parameters, the bifurcation point may be shifted to a location in the combined space that does not allow for bistability. This dynamic behaviour is an emergent property of the system as a whole and would not have easily been predicted from the gating behaviour of the sodium channels alone.

Combining empirical measurements of impairments in molecular function with computational neuronal modelling integrates different scales of evidence. A similar approach identified novel pathological mechanisms resulting in abnormally increased persistent sodium currents through mutant channels in *SCN1A* mutations^32^. Computational models have helped identify a common mechanism of epilepsy pathophysiology: apparently functionally disparate mutations affecting different sodium channels can result in neuronal hyperexcitability^33^.

This has implications for further improvements of antiepileptic drug choice in Dravet syndrome. The clinical effectiveness of valproate in our patient is in keeping with our results and valproate’s effect on inducing use-dependent limitations on fast action potential firing^34^. However, valproate can have significant adverse effects, and is not effective in all patients. Currently, sodium channel blockers such as carbamazepine are avoided in standard clinical practice because of frequent reports of associated paradoxical worsening seizures^35^. Detailed knowledge of mutation locus and associated gating abnormalities may help stratify the risk of adverse effects and permit the addition of an anti-epileptic drug with direct sodium channel blocking action for some patients. One example is lidocaine, which is already used in some centres for the treatment of status epilepticus^36^. It binds directly to the S4 voltage-sensing subunits of the channel and particularly stabilises the S4 in DIII in a depolarised condition^37^. Furthermore, it binds preferentially to fast-inactivated channel conformations^38^. It may therefore be a valuable addition to treatment protocols specifically for patients with similar dynamic gating abnormalities in a high-frequency firing state (e.g. status epilepticus) and may warrant further detailed study in whole organism models of such *SCN1A* mutations.

Our study design allows for comprehensive assessment of the molecular functional effects of the mutation under different, controlled experimental conditions. Describing the dynamic effects caused directly by the mutation through a computational model yielded a novel mechanism of seizure susceptibility for this epilepsy patient, consistent with his phenotype.

Using these functional evaluations to achieve clinical improvements will require further study. Functional neurophysiological studies combined with computational modelling, illustrated in this case, show that rich information can be derived about abnormal neuronal dynamics for individual patients. Conducting these experiments on a larger scale will confirm whether mechanisms observed in individual patients are unique or will converge to common mechanisms that correlate to the phenotypes.

## Methods

### Bacterial Transformations and Mutagenesis

The patient’s mutation was identified through bidirectional Sanger sequencing by the West of Scotland Genetic Services laboratories as part of routine clinical workup. The use of genetic and clinical information for this study and all experimental protocols were approved by the UK National Research Ethics Service (NRES). Written consent was given by the patient’s parents prior to the experiments described below. Use of all clinical and genetic information was carried out in accordance with relevant guidelines (including university and hospital data protection guidelines, as well as the principles laid out in the Good Clinical Practice framework).

All bacterial transformations for this project were performed in TOP10/P3 *E. coli* bacteria (Invitrogen). As *SCN1A* is known to spontaneously mutate in bacterial cultures, the entire length of the sodium channel was sequenced following every transformation. The original *SCN1A* DNA in the PCDM8 vector was graciously provided by Dr. Lori Isom (University of Michigan). All transformants from the original *SCN1A* vector were found to express the T1967A and G5923T mutations (compared to NM_006920.4), which encode the V650E and A1969S mutants in Na_V_1.1, respectively. A QuikChange Lightning Mutagenesis kit (Agilent Technologies, CA, USA) with the following primers was used to get the WT and mutant DNA.

**A1967T**

5' – CACTGTGGATTGCAATGGTG**T**GGTTTCCTTGGTTGGTGGAC-3'

5' – GTCCACCAACCAAGGAAACC**A**CACCATTGCAATCCACAGTG-3'

**T5923G**

5' – CTGATCTGACCATGTCCACT**G**CAGCTTGTCCACCTTCC-3'

5' – GGAAGGTGGACAAGCTG**C**AGTGGACATGGTCAGATCAG-3'

**C3818T**

5' – CTGGAAATGCTTCTAAAATGGGTGG**T**ATATGGCTATCAAAC-3'

5' – GTTTGATAGCCATAT**A**CCACCCATTTTAGAAGCATTTCCAG – 3'

### Electrophysiology

We grew CHOk1 cells (Sigma-Aldrich, MO, USA) in Ham’s F12 medium supplemented with 10% FBS at 37C in 5% CO2. 24–48 hours prior to experimentation we used Polyfect transfection reagent (Qiagen, Venlo, NL) to transfect cells with 1ug of the *SCN1A*, 1ug of eGFP, and 0.5 ug of the β1 subunit using the protocols suggested by Qiagen. 8–12 hours after transfection, we plated cells on sterile glass coverslips.

Whole cell patch clamp experiments were performed at 32 °C, 37 °C, and 40 °C using borosilicate glass pipettes pulled with a P-1000 puller (Sutter Instruments, CA, USA), dipped in dental wax, and polished to a resistance of 1.0–1.5 MΩ. Extracellular solutions contained (in mM): 140 NaCl, 4 KCl, 2 CaCl_2_, 1 MgCl_2_, and 10 HEPES. Intracellular solutions contained (in mM): 130 CsF, 10 NaCl, 10 HEPES, and 10 EGTA. We titrated extracellular and intracellular solutions to pH 7.4 with CsOH.

We performed all experiments using an EPC9 patch-clamp amplifier digitized using an ITC-16 interface (HEKA Elektronik, Lambrecht, Germany). For data collection and analysis we used Patchmaster/Fitmaster (HEKA Elektronik) and Igor Pro (Wavemetrics, OR, USA) running on an iMac (Apple Inc., CA, USA). Temperature was maintained using a TC-10 temperature controller (Dagan Corporation, MN, USA). We low-pass-filtered the data at 5kHz and used a P/4 leak subtraction procedure for all recordings. The holding potential between protocols was −90 mV.

### Pulse Protocols and Analysis

Macroscopic currents were elicited with 20 ms depolarizations to membrane potentials between −100 mV and +60 mV. Conductance was determined by dividing peak current by the experimentally observed reversal potential subtracted from membrane potential. Normalized conductance plotted against voltage was fit by a single Boltzmann equation. The decay of current was fit by a single exponential equation to determine the time constant of open state inactivation at a given voltage.

Steady-state fast inactivation was measured as the proportion of current remaining in a test pulse to 0 mV mV following 200 ms pulses to voltages between −130 mV and +10 mV. The normalized current plotted against voltage was fit by a single Boltzmann equation.

The time course of fast inactivation recovery at −90 mV was measured as the proportion of current after a 200 ms depolarization to 0 mV and a recovery pulse of varying lengths to −90 mV. The normalized current was plotted versus recovery time and fit with a double exponential equation.

### Statistical Analysis

We used a two-factor, completely random design analysis of variance to test first for evidence that the A1273V mutant has differential temperature sensitivity compared to the WT channel. The mutant effect was evaluated as a nominal independent variable and temperature as a continuous independent variable. The distribution of residuals from the statistical model were approximately normally distributed. In this analysis, the interaction between temperature (continuous variable) *vs*. mutant (nominal variable), was used as a predictor variable. A significant difference in the interaction term was evidence of a difference in temperature sensitivity between the mutant and WT channels. If this was not the case, then the analysis was repeated without the interaction term to test for the main mutant and temperature effect. All statistical analyses were performed using JMP software (SAS Institute, NC, USA). Statistical significance was evaluated at P < 0.05. and measurements of error are reported as standard error of the mean. Means, standard error of the mean, and n values for experimental data are reported in the source data contained in Supplementary Tables S1–S5. P-values for a given statistical test are mentioned in text where appropriate.

### Modelling

Mutation effects on action potential generation were modelled using Hodgkin-Huxley (HH) models adapted to fit the dynamics of responses seen in regular spiking cortical pyramidal cells^39^. The full model is available online (https://github.com/roschkoenig/SCN1A_HodgkinHuxley), together with an explanation how to derive the model-related figures in this manuscript. The HH model estimates changes in membrane potential from non-linear, voltage-dependent changes in ion-specific membrane conductances:


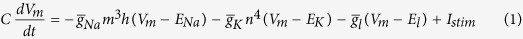



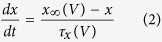



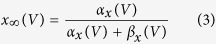



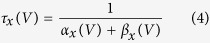


where *x* is *m*, *n*, or *h*


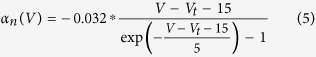



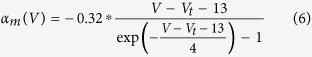



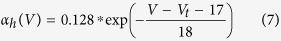



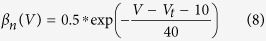



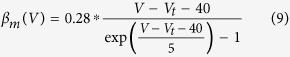



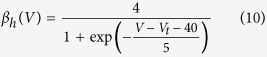


The equations represent a system of coupled ordinary differential equations describing the changes of membrane voltage (Eq. 1) and ion channel gating parameters (Eq. 2). In this formulation there are only two ion channels — voltage gated-potassium channels, and voltage-gated sodium channels — the latter of which are of particular interest as we aim to parameterise a model that implements the experimentally derived changes in gating parameters *m* and *h* for the voltage gated sodium channel to estimate the resultant abnormal dynamics of the neuronal membrane.

At steady state, gating parameters are described by Eq. 3^40^. This represents a sigmoid function, which can also be parameterised using the generic Boltzmann formulation^41^, Eq. 11:


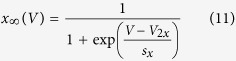


Experimental measurements taken from voltage clamp experiments include measures of midpoints (*V*_*2x*_) and slope (s_x_) of conductance and fast inactivation of sodium channels, representative of steady state gating parameters *m* and *h* respectively, and thus speak naturally to the Boltzmann formulation of gating parameters. However, because the experimental design did not fully replicate physiological states in cortical neurons (as a different cellular model was used, and non-physiological additives such as fluoride are used experimentally to stabilise the membrane), we normalised our results to those described in^39^ as follows:

1. Simulate cortical neurons using the parameterisation of^39^.
2. Fit Boltzmann equations parameters to these simulations and derive midpoint voltages and slope of steady state gating parameters for the Pospischil parameterization.
3. For the WT simulations at 37 °C, the parameters (V_2m_, s_m_, V_2h_, s_h_) were set to the same values as for the Pospischil parameterization.
4. For all remaining simulations, parameters were adjusted using the original parameterisation as baseline – preserving the absolute offset compared to the WT (V_2m_, V_2h_), or relative difference compared to the WT (s_m_, s_h_).
5. An additional offset parameter was introduced for fast inactivation gating equations 7 and 10 to account for temperature-dependent differences in time constants (note that with the Boltzman formulation for equation 3, only time constants (Eq. 4) depend on equations 5, 6, 7, 8, 9, 10).

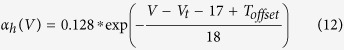



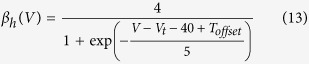


This yields four parameterisations of the same model, which represent different degrees of deviations from a standard model of regular spiking pyramidal cells. This normalisation was introduced to ensure that our experimental values are translated into physiological parameter spaces, but preserve the relative differences of the parameters between experimental conditions. Parameter values used for capacitance, channel conductances, and Nernst potentials are summarised in Table 1.

We examined the four versions of the model in terms of their response to different levels of stimulation (*I*_*stim*_, Eq 1). This was first performed qualitatively and then further assessed using systematic variations and bifurcation analysis.

## Additional Information

**How to cite this article**: Peters, C. *et al*. Temperature-dependent changes in neuronal dynamics in a patient with an *SCN1A* mutation and hyperthermia induced seizures. *Sci. Rep.* **6**, 31879; doi: 10.1038/srep31879 (2016).

## Supplementary Material

Supplementary Information

## Figures and Tables

**Figure 1:**
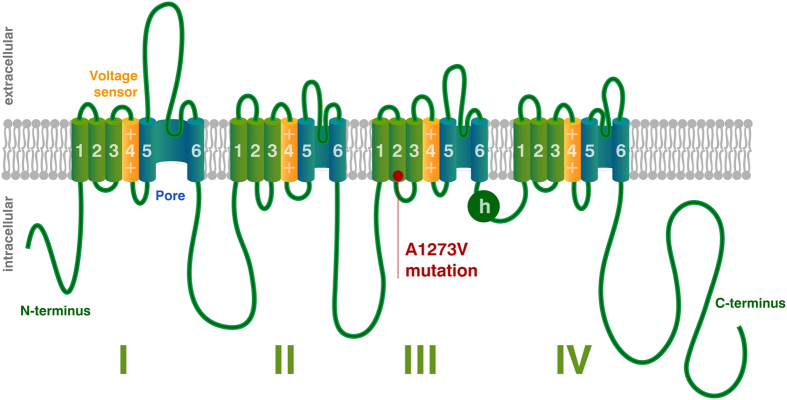
Schematic Sodium Channel Topology. Schematic showing the membrane structure of mammalian voltage-gated sodium channels. The pore-forming regions of each domain are shown in blue while the positively charged S4 voltage-sensing segments are shown in yellow. The locations of the fast inactivation particle (h gate) and the A1273V mutant are also shown.

**Table 1:** Parameter values for the computational model in the four different conditions evaluated.

Parameter	Units	WT37	WT40	AV37	AV40
C	*μA/mm*^*2*^	0.010	0.010	0.010	0.010
g_L_	*μS/cm*^*2*^	0.0205	0.0205	0.0205	0.0205
g_K_	*mS/cm*^*2*^	5	5	5	5
g_Na_	*mS/cm*^*2*^	56	56	56	56
E_L_	*mV*	−70.3	−70.3	−70.3	−70.3
E_K_	*mV*	−90	−90	−90	−90
E_Na_	*mV*	50	50	50	50
I_stim_	*μA/mm*^*2*^	0.20	0.20	0.20	0.20
V_t_	*mV*	−55	−55	−55	−55
**s**_**m**_	—	**7.4**	**6.6**	**6.1**	**7.6**
**s**_**h**_	—	**−4.0**	**−3.4**	**−3.4**	**3.8**
**V**_**2m**_	***mV***	**−39.0**	**−32.5**	**−33.1**	**−29.1**
**V**_**2h**_	***mV***	**−43.3**	**−45.2**	**−41.0**	**−31.6**
**T**_**off**_	***mV***	**0**	**1.9**	**−2.4**	**−11.7**

All parameters were normalized to an existing model of cortical Hodgkin-Huxley dynamics, and empirical differences represented as appropriate shifts in the model parameters. The whole code to reproduce the figures relating to computational modelling in this manuscript is available online: github.com/roschkoenig/SCN1A_HodgkinHuxley.
