# Supplementary material for: Temperature-dependent changes in neuronal dynamics in a patient with an SCN1A mutation and hyperthermia induced seizures
Source: Sci Rep. 2016 Sep 1;6:31879. doi: 10.1038/srep31879 (PMC5007485; doi:10.1038/srep31879)
Supplement: Supplementary Information [file srep31879-s1.pdf]

Temperature-dependent changes in neuronal dynamics in a patient with an *SCN1A* mutation and hyperthermia induced seizures

Peters C[1*], Rosch RE[2,3*], Hughes E[4], Ruben P[1]

[1]Department of Biomedical Physiology and Kinesiology, Simon Fraser University, Burnaby, BC, Canada

[2]Wellcome Trust Centre for Neuroimaging, Institute of Neurology, University College London, UK

[3]Centre for Developmental Cognitive Neuroscience, Institute of Child Health, University College London, UK

[4]Department of Paediatric Neurology, Evelina London Children's Hospital, Guy's and St Thomas' NHS Foundation Trust, London, UK

[*] Equal Contribution

Supplementary Material:

Supplementary Table S1

	**WT 32°C**			**AV 32°C**		
	**Mean**	**StErr**	**N**	**Mean**	**StErr**	**N**
**G V½ (mV)**	-12.6	2.1	11	-16.4	1.9	8
**G z**	3.73	0.28	11	3.62	0.18	8
**SSFI V½ (mV)**	-50.2	2.6	9	-58.9	1.6	9
**SSFI z**	-4.07	0.30	9	-3.12	0.37	9
**FIrec τ1 (ms)**	2.18	0.29	9	2.55	0.70	5
**FIrec A1**	0.597	0.030	9	0.508	0.046	5
**FIrec τ2 (ms)**	97	20	9	125	34	5
**FIrec A2**	0.357	0.024	9	0.400	0.025	5
**-10mV, FIon τ (ms)**	0.641	0.097	12	0.571	0.104	7
**0mV, FIon τ (ms)**	0.364	0.043	12	0.327	0.030	7
**10mV, FIon τ (ms)**	0.256	0.026	12	0.246	0.028	7
**20mV, FIon τ (ms)**	0.181	0.014	12	0.178	0.027	7
**30mV, FIon τ (ms)**	0.163	0.015	12	0.176	0.028	6
**40mV, FIon τ (ms)**	0.154	0.016	11	0.140	0.015	5
**-20mV, 50% Act (ms)**	0.303	0.011	11	0.304	0.016	9
**-10mV, 50% Act (ms)**	0.290	0.010	12	0.300	0.013	9
**0mV, 50% Act (ms)**	0.274	0.010	12	0.277	0.013	9
**10mV, 50% Act (ms)**	0.256	0.011	12	0.261	0.013	9
**20mV, 50% Act (ms)**	0.238	0.011	12	0.244	0.012	9
**30mV, 50% Act (ms)**	0.224	0.010	12	0.228	0.013	8
**40mV, 50% Act (ms)**	0.219	0.014	10	0.214	0.012	9
**50mV, 50% Act (ms)**	0.211	0.013	10	0.210	0.013	9
**60mV, 50% Act (ms)**	0.209	0.012	10	0.194	0.011	5

Table S1: Averages and standard errors of data at 32°C.
Conductance (G) and steady-state fast inactivation (FI) curves were fit with Boltzmann curves; V½ and z are the midpoint and apparent valence, respectively. Fast inactivation recovery curves (FIrec) at -90mV were fit with a double exponential equation; τ1 and A1 are the fast time constant and fast amplitude, respectively, while τ2 and A2 are the slow time constant and slow amplitude, respectively. Decay of macroscopic currents were fit with a single exponential equation to measure the time constant of open-state inactivation (FIon τ). The times between the voltage clamp of membrane potential and the time of 50% maximal current at a given membrane potential were used as measures of activation (50% Act).

Supplementary Table S2:

	NaV1.1, WT, 37°C			NaV1.1, A1273V, 37°C		
	**Mean**	**StErr**	**N**	**Mean**	**StErr**	**N**
**-20mV, 50% Act (ms)**	0.269	0.057	9	0.267	0.011	5
**-10mV, 50% Act (ms)**	0.250	0.008	9	0.266	0.008	5
**0mV, 50% Act (ms)**	0.227	0.006	9	0.257	0.012	5
**10mV, 50% Act (ms)**	0.211	0.005	9	0.237	0.012	5
**20mV, 50% Act (ms)**	0.199	0.004	9	0.218	0.011	5
**30mV, 50% Act (ms)**	0.191	0.004	9	0.204	0.010	5
**40mV, 50% Act (ms)**	0.185	0.004	9	0.194	0.009	5
**50mV, 50% Act (ms)**	0.181	0.006	6	0.187	0.008	5
**60mV, 50% Act (ms)**	0.182	0.006	6	0.180	0.010	5
	**NaV1.1, WT, 40°C**			**NaV1.1, A1273V, 40°C**		
**-20mV, 50% Act (ms)**	0.260	0.023	4	0.263	0.009	9
**-10mV, 50% Act (ms)**	0.261	0.019	7	0.270	0.010	10
**0mV, 50% Act (ms)**	0.238	0.016	7	0.256	0.011	10
**10mV, 50% Act (ms)**	0.222	0.015	7	0.239	0.012	10
**20mV, 50% Act (ms)**	0.206	0.013	7	0.225	0.010	10
**30mV, 50% Act (ms)**	0.196	0.011	7	0.211	0.009	10
**40mV, 50% Act (ms)**	0.189	0.010	7	0.200	0.005	10
**50mV, 50% Act (ms)**	0.182	0.008	7	0.183	0.006	8
**60mV, 50% Act (ms)**	0.181	0.013	6	0.186	0.007	8

Source Data for Figure 1 C and D:
The times between the voltage clamp of membrane potential and the time of 50% maximal current at a given membrane potential were used as measures of activation (50% Act).

Supplementary Table S3:

	NaV1.1, WT, 37°C			NaV1.1, A1273V, 37°C		
	**Mean**	**StErr**	**N**	**Mean**	**StErr**	**N**
**G V½ (mV)**	-14.1	3.1	9	-12.0	1.5	5
**G z**	3.43	0.24	9	4.08	0.21	5
**SSFI V½ (mV)**	-57.2	1.1	5	-54.8	3.7	5
**SSFI z**	-3.14	0.11	5	-3.73	0.63	5
	**NaV1.1, WT, 40°C**			**NaV1.1, A1273V, 40°C**		
**G V½ (mV)**	-10.0	2.0	7	-4.2	2.0	11
**G z**	3.39	0.19	7	3.07	0.19	11
**SSFI V½ (mV)**	-59.1	3.4	5	-45.5	3.1	6
**SSFI z**	-3.79	0.17	5	-3.38	0.49	6

Source Data for Figure 2:

Conductance (G) and steady-state fast inactivation (FI) curves were fit with Boltzmann curves; V½ and z are the midpoint and apparent valence, respectively.

Supplementary Table S4:

	NaV1.1, WT, 37°C			NaV1.1, A1273V, 37°C		
	**Mean**	**StErr**	**N**	**Mean**	**StErr**	**N**
**FIrec τ1 (ms)**	1.62	0.44	5	1.42	0.23	5
**FIrec A1**	0.542	0.087	5	0.536	0.041	5
**FIrec τ2 (ms)**	81	16	5	141	56	5
**FIrec A2**	0.418	0.022	5	0.337	0.023	5
	**NaV1.1, WT, 40°C**			**NaV1.1, A1273V, 40°C**		
**FIrec τ1 (ms)**	1.27	0.21	6	1.24	0.13	5
**FIrec A1**	0.396	0.030	6	0.623	0.033	5
**FIrec τ2 (ms)**	72	22	6	138	57	5
**FIrec A2**	0.503	0.034	6	0.285	0.033	5

Source Data for Figure 3 A and B:

Fast inactivation recovery curves (FIrec) at -90mV were fit with a double exponential equation; τ1 and A1 are the fast time constant and fast amplitude, respectively, while τ2 and A2 are the slow time constant and slow amplitude, respectively.

Supplementary Table S5:

	NaV1.1, WT, 37°C			NaV1.1, A1273V, 37°C		
	**Mean**	**StErr**	**N**	**Mean**	**StErr**	**N**
**-10mV, FIon τ (ms)**	0.369	0.040	6	0.809	0.102	5
**0mV, FIon τ (ms)**	0.249	0.024	6	0.429	0.017	5
**10mV, FIon τ (ms)**	0.181	0.017	6	0.273	0.013	5
**20mV, FIon τ (ms)**	0.188	0.022	6	0.209	0.017	5
**30mV, FIon τ (ms)**	0.168	0.023	6	0.165	0.026	5
**40mV, FIon τ (ms)**	0.134	0.010	5	0.158	0.033	5
	**NaV1.1, WT, 40°C**			**NaV1.1, A1273V, 40°C**		
**-10mV, FIon τ (ms)**	0.494	0.100	7	0.686	0.117	9
**0mV, FIon τ (ms)**	0.259	0.042	7	0.487	0.104	9
**10mV, FIon τ (ms)**	0.170	0.026	7	0.245	0.037	9
**20mV, FIon τ (ms)**	0.159	0.025	7	0.174	0.025	9
**30mV, FIon τ (ms)**	0.127	0.016	7	0.150	0.014	9
**40mV, FIon τ (ms)**	0.124	0.017	7	0.116	0.010	6

Source Data for Figure 3 C and D:
Decay of macroscopic currents were fit with a single exponential equation to measure the time constant of open state fast inactivation at a given membrane potential.

Supplementary Figure S1:

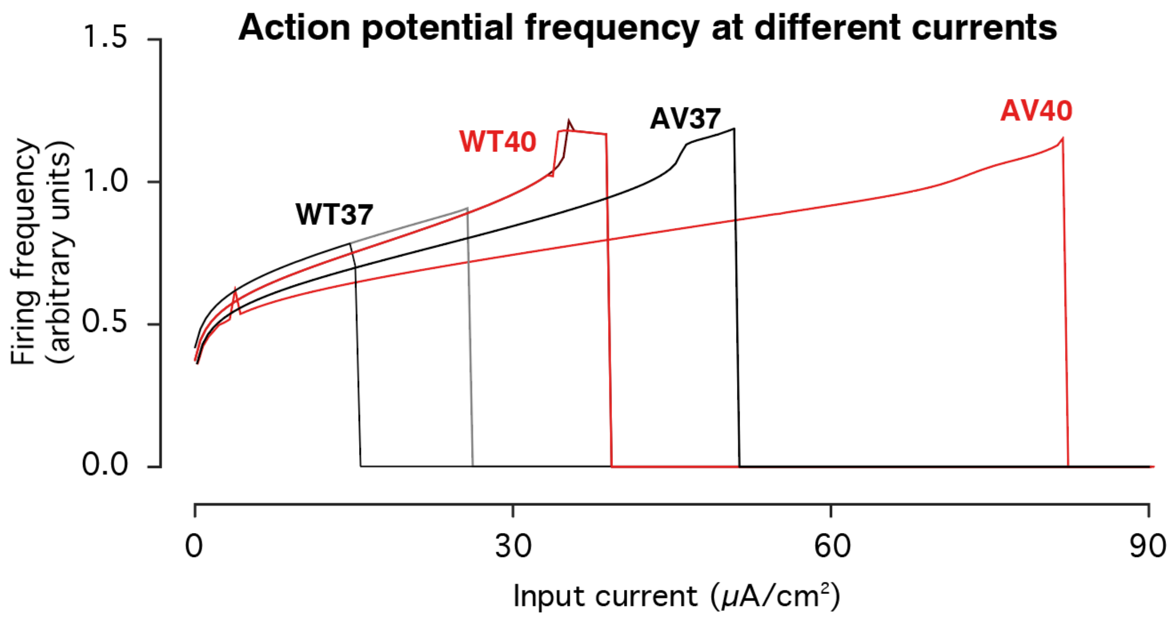

Modelled Action Potential Firing Frequency:
Action potential frequency is shown for the adapted Hodgkin-Huxley models for each of the four experimental conditions (i.e. wild type at 37ºC [WT37] and 40ºC [WT40], mutation at 37ºC [AV37] and 40ºC [AV40]). Modelling for all condition was performed in two directions (i.e. increasing, and decreasing values of stimulation currents), revealing hysteresis in the WT at 37ºC only (grey – increasing stimulating currents; black – decreasing stimulating currents).
The plot shows the temperature-sensitive increase in stimulation tolerance across conditions. However, for any given stimulation current the predicted action potential frequency is lower for the low temperature vs high temperature conditions.
